# The Effects of Nature-Based Travel in Virtual Reality: The Role of Spatial Presence and Narrative Engagement

**DOI:** 10.1089/cyber.2022.0240

**Published:** 2023-09-11

**Authors:** Christopher Ball

**Affiliations:** Department of Journalism, Institute of Communications Research, University of Illinois at Urbana-Champaign, Urbana, Illinois, USA.

**Keywords:** virtual reality, nature tourism and travel, environment, spatial presence, narrative engagement

## Abstract

The benefits of nature tourism, or nature-based travel, are plentiful. *For example*, participation in nature tours has positively impacted environmental attitudes and behaviors. Unfortunately, while psychologically beneficial, nature-based tourism can hurt the environment through a myriad of factors. Therefore, we must continue to explore ways to make the benefits of nature-based travel more sustainable and impactful. Research suggests that nature-based travel in virtual reality (VR) may impart numerous travel benefits, such as improving conservational behavior and interconnectedness with nature. While these early findings are promising, questions remain regarding the theoretical mechanisms underlying the effects of nature-based VR travel. Therefore, this study explores how VR may provide an avenue to make nature tourism more environmentally friendly while simultaneously making people more environmentally connected and conscious. Furthermore, a theoretical framework is posited that combines concepts from the spatial presence and narrative persuasion literature to help explain the effects. To accomplish these goals, an experiment was conducted using a two-condition (VR travel vs. TV control) between-subjects factorial design with random assignment. The participants were 66 college students from a large Midwestern University in the United States. Results indicated that there wasn't a statistically significant difference between the VR travel condition and the television (TV) control condition regarding the environmental outcome variables. However, while the nature-based VR travel experience did not appear to influence the environmental outcome variables directly, it did indirectly affect them through the mediating roles of spatial presence and narrative engagement.

## Introduction

The benefits of nature tourism, or nature-based travel, are plentiful. *For example*, participation in nature tours has positively impacted environmental attitudes and behaviors.^1^ Nature tourism is also associated with increased conservation behavioral intentions regarding local conservation issues.^2^ Unfortunately, while psychologically beneficial, tourism can hurt the environment through a myriad of factors such as increased carbon emissions.^3^ Paradoxically, nature tourism, in particular, has been found to hurt the very natural environments we seek to connect with.^4^ Therefore, we must continue to explore ways to make the benefits of nature-based travel more sustainable and impactful. The present study explores how virtual reality (VR) may provide a new avenue to make nature tourism more environmentally friendly while simultaneously making people more environmentally connected and conscious.

The impacts of VR on travel have been speculated for decades.^5^ However, with the recent introduction of consumer-grade VR head-mounted displays (HMDs), we have seen increased interest from academics and consumers alike. *For example*, VR tourism/travel was one of the most reported uses of VR during the COVID-19 pandemic.^6^ Importantly, early research suggests that there are many potential benefits to nature-based VR travel. *For example*, experiencing a nature-based 360° video positively impacted participants' conservational behavior intentions.^7^ Furthermore, compared to a print condition, participants in a pro-environmental VR experience were found to demonstrate increased pro-environmental behaviors.^8^ Likewise, compared to a video condition, a pro-environmental VR experience increased participants' feelings of interconnectedness with nature.^9^ Therefore, VR nature-based travel experiences may provide a new avenue for us positively to impact the environment directly (i.e., by reducing carbon emissions) and indirectly (i.e., by improving environmental attitudes and behavioral intentions).

While these early findings are promising, questions remain regarding the theoretical mechanisms that could explain the effects of nature-based VR travel. *For example*, one study found little difference between watching a nature-based 360° video in VR or on a tablet.^10^ The present study explores the potential of a nature-based VR travel experience to persuade people to become more environmentally connected and conscious. Furthermore, this study examines the potential theoretical mechanisms that may underlie the effects of nature-based VR travel. Specifically, an experiment is conducted to explore the impacts of nature-based VR travel on spatial presence and narrative engagement and their role as potential mediators. To accomplish these goals, a two-condition (VR vs. TV) between-subjects factorial design experiment was conducted with a sample of college students.

### VR, spatial presence, and narrative engagement

Spatial presence and narrative engagement have been frequently discussed in VR Research. Definitions of spatial presence vary, but for this study, it is defined as the cognitive perception that a person's body is occupying a space in which they feel in sync with the actions of the virtual experience.^9,11,12^ The immersive features of VR experiences have been linked to higher reported feelings of presence compared to traditional media.^13^ *For example*, studies have found that participants reported greater feelings of presence in VR compared to desktop computer conditions during both science laboratory simulations and earthquake simulations.^14,15^ Therefore, we posit that those experiencing nature in VR versus a TV will report greater levels of spatial presence. Spatial presence has also been found to mediate the impact of pro-environmental VR experiences on environmental variables, such as inclusion of nature in self.^9^ Thus, a VR nature experience may result in greater feelings of being connected to nature.

Narrative persuasion is “any influence on beliefs, attitudes, or actions brought about by a narrative message through processes associated with narrative comprehension or engagement”.^16^ One critical factor that influences a narrative's ability to have a persuasive impact is narrative engagement.^17^ Interactive virtual experiences, such as those provided by VR, can result in greater immersion within a narrative.^18^ Therefore, those who experience a VR nature narrative should report greater feelings of narrative engagement compared to traditional media. Furthermore, a person that is more engaged with a narrative is more likely to be persuaded or have their attitudes changed by its message.^19^ Thus, if a person is engaged with an ocean related VR narrative their ocean protection attitudes may improve as a result. Finally, participants in a virtual experience containing a narrative had higher heart rates and motivation to complete a task compared to the same experience without a narrative context.^20^ Therefore, a virtual nature-based experience may result in higher levels of behavioral intentions such as support for conservation and activism intentions.

**FIG. 1.**
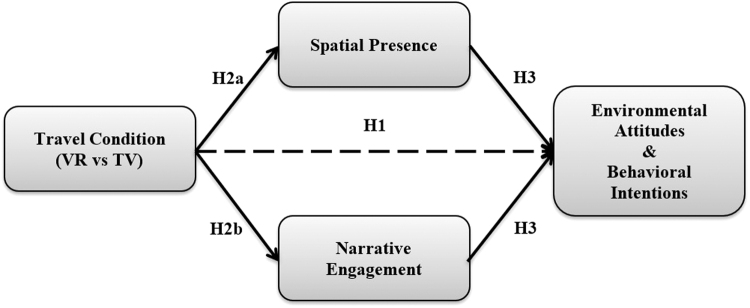
Proposed theoretical framework. VR, virtual reality.

Therefore, in the present study, spatial presence is conceptualized as feeling present within the *context* of a virtual experience (i.e., the place), while being narratively engaged is being engaged with the *content* of a virtual experience (i.e., the experience/story). Figure 1 represents the theoretical framework proposed in this study, and the present study seeks to test the following hypotheses:

**H1: Participants in the VR travel condition will report higher levels of (a) connection to nature, (b) support for conservation, (c) activism intentions, and (d) ocean protection attitudes compared to those in the TV travel control condition.**

**H2: Participants in the VR travel condition will report higher levels of (a) spatial presence and (b) narrative engagement than those in the low immersion (TV) condition.**

**H3: Spatial presence and narrative engagement will mediate the relationship between travel conditions and (a) connection to nature, (b) support for conservation, (c) activism intentions, and (d) ocean protection attitudes.**

## Methods

### Participants

To test the above hypotheses, an institutional review board-approved experiment was conducted using a sample of college students. Specifically, this study used a two-condition (VR vs. TV) between-subjects factorial design. Participants were randomly assigned to each condition. Participants were 66 college students (34 in the VR condition and 32 in the TV condition) drawn from a large Midwestern University in the United States. An *a priori* power analysis was conducted using G Power based on the moderate effect size found in a prior study which examined the mediating role of spatial presence on inclusion of nature in self (*b* = 0.67, α = 0.04).^9^ Results of the power analysis indicated the need for a sample size of at least 62 to detect such an effect size which would surpass the 0.80 power threshold.^21,22^ Recruitment was conducted through an interdepartmental research subject pool. Students were given one extra credit point (1 percent of their final grade) for participating in this study as an incentive. Approximately 70 percent of participants were female, 60 percent were white, and the average age was 20.

### Materials

The VR travel stimulus used in this study is called *theBlu*,^23^ which contains several short experiences in various underwater environments. In this study, the “whale encounter” experience was selected. During the whale encounter experience, participants find themselves on the deck of a sunken ship with various wildlife (i.e., fish, manta rays, and a humpback whale) swimming around the ship and the participant. *theBlu* is an entirely passive experience that does not require any actions from the player to experience, and it takes ∼2 minutes to complete. The stimulus was experienced using an HTC Vive HMD in the VR condition or viewing it on a 19″ high-definition computer monitor in the control condition. The HTC Vive VR equipment included HMDs, two infrared tracking base stations, and two motion controllers. The study occurred in a laboratory with only a desk, a chair, the necessary VR-related equipment, and no other distracting elements.

### Procedure

When the participants arrived at the laboratory, they completed a pretest questionnaire that measured their baseline environmental attitudes and behavioral intentions and gathered demographic information. Participants assigned to the VR travel condition were then instructed on how to use the Vive equipment. Participants assigned to the TV control condition were simply asked to watch a short 2D video version of the *theBlu* whale encounter stimulus. In either case, after the stimulus period concluded, the post-test questionnaire was administered, which included the spatial presence and narrative engagement measures. Regardless of the participant's completion of the study, all participants were debriefed, and their participation incentives were dispensed.

### Measures

Spatial presence was measured using the Spatial Presence Experience Scale (SPES).^12^ The SPES measures spatial presence across two dimensions: self-location and possible actions. The two dimensions form an 8-item scale (α = 0.92). Narrative engagement, a factor needed for narrative persuasion, was measured using a modified version of the Narrative Engagement Scale (NES).^17^ The NES measures narrative engagement across four dimensions: attentional focus, narrative presence, emotional engagement, and narrative understanding. The subconstruct of “narrative understanding” was omitted from this study because it was tied directly to traditional narratives (written stories), and *the* stimulus does not have a conventional narrative structure. The resulting was 7-item scale (α = 0.73).

Environmental attitudes and behavioral intentions were measured across four variables. Connectedness to nature was measured using the Connectedness to Nature Scale (CNS).^24^ The CNS is a 14-item scale (α = 0.86). Support for conservation and environmental activism intentions were measured using scales from the Environmental Attitude Inventory.^25^ The support for conservation scale contains 10 items (α = 0.87). The environmental activism intentions scale also contains 10 items (α = 0.91). Finally, ocean protection attitudes were measured using questions from The Ocean Project Tracking Survey (OPTS).^26^ A selection of 13 relevant survey items was drawn from the OPTS, and then an exploratory factor analysis was conducted using varimax rotation. A distinct factor emerged with strong loadings (>0.8) and no significant cross-loadings (<0.4). The resulting 3-item scale (α = 0.86) contained questions related to the protection of the world's oceans. The above scale items were measured using a 5-Point Likert-like scale (1 = strongly disagree and 5 = strongly agree). The scale items were totaled and then averaged to produce overall variable scores.

## Results

### Independent samples *t* tests: H1 and H2

A set of independent samples *t* tests were conducted to test the first two hypotheses. The first hypothesis (H1) predicted that students in the VR travel condition would report higher environmental attitudes and behavioral intentions than those in the TV control condition. In this case, there were no statistically significant differences between the experimental and control groups regarding any of the environmental attitude variables.

The mean scores for connectedness to nature compared between the experimental group (*M* = 3.69, *SD* = 0.73) and control group (*M* = 3.77, *SD* = 0.53) were not statistically significantly different [*t*(64) = 0.50, *p* = 0.62]. The mean scores for support for conservation compared between the experimental (*M* = 4.16, SD = 0.71) and control groups (*M* = 4.11, *SD* = 0.65) were not statistically significantly different [*t*(64) = −0.30, *p* = 0.77]. The mean scores for activism intentions compared between the experimental (*M* = 3.76, *SD* = 0.84) and control groups (*M* = 3.76, *SD* = 0.78) were not statistically significantly different [*t*(64) = 0.01, *p* = 0.99]. Finally, the mean scores for ocean protection attitudes compared between the experimental (*M* = 4.43, *SD* = 0.83) and control groups (*M* = 4.46, *SD* = 0.61) were also not statistically significantly different [*t*(64) = 0.15, *p* = 0.88]. Therefore, H1 was not supported.

The second hypothesis (H2a) predicted that students in the VR travel condition would report higher levels of spatial presence than those in the TV control condition. The *t* test results indicated that there was a statistically significant difference in spatial presence scores between the experimental and control groups [*t*(62) = −3.85, *p* < 0.001]. The mean spatial presence scores for the VR travel condition were significantly higher (*M* = 4.29, *SD* = 0.76) than those in the TV control condition (*M* = 3.52, *SD* = 0.86). Likewise, the second hypothesis (H2b) predicted that students in the VR travel condition would report higher levels of narrative engagement than those in the TV control condition. Once again, the *t* test results indicated a statistically significant difference in the mean narrative engagement scores between the experimental and control groups [*t*(54) = −2.74, *p* = 0.008]. The mean scores for narrative engagement were significantly higher (*M* = 3.89, *SD* = 0.57) for the VR condition than those of the TV control condition (*M* = 3.40, *SD* = 0.86). Therefore, H2a and H2b were supported.

### Mediation analysis: H3

**FIG. 2.**
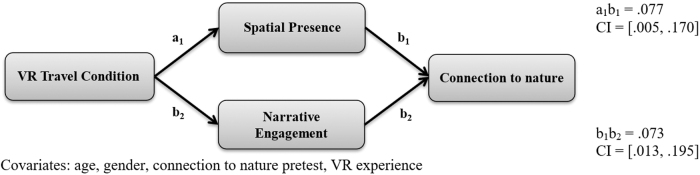
Indirect effect of VR travel condition on connection to nature. CI, confidence interval.

The mediation analysis was conducted using the PROCESS path-analysis SPSS macro (Model 4) employing a boot-strapping method of 5,000 samples.^27^ First, a parallel mediation analysis examined connectedness to nature (Table 1 and Fig. 2). The VR travel condition positively predicted spatial presence (*b* = 0.84, *p* < 0.001) and narrative engagement (*b* = 0.52, *p* < 0.01). Narrative engagement was a statistically significant predictor of connectedness to nature (*b* = 0.14, *p* < 0.05) while spatial presence was approaching statistical significance (*b* = 0.09, *p* < 0.10). The indirect effect from VR travel condition to spatial presence and then to connectedness to nature was statistically significant (confidence interval [95% CI 0.005–0.17]). The indirect effect from VR travel condition to narrative engagement and then to connectedness to nature was also statistically significant (95% CI [0.013–0.195]). Therefore, H3a was supported. The mediation model explained a statistically significant proportion of variance in connectedness to nature scores [*R*^2^ = 0.85, *F*(8, 57) = 41.24, *p* < 0.001].

**Table 1.** Mediation/Regression Results Showing Direct and Indirect Effects of Virtual Reality Travel Condition on Connectedness to Nature and Support for Conservation

	Connectedness to nature			Support for conservation		
	Coeff.		SE	Coeff.		SE
Direct effects						
Spatial presence	0.84	^***^	0.19	0.80	^***^	0.21
Narrative engagement	0.52	^**^	0.19	0.50	^**^	0.18
Indirect effects						
Spatial presence	0.09	^+^	0.05	0.12	^**^	0.05
Narrative engagement	0.14	^**^	0.05	0.01		0.06
*F*	41.24	^***^		41.00	^***^	
*R* ^2^	0.85			0.85		

*Note:* age, gender, race, VR experience, and pretest results were controlled.

*N* = 66; ^+^*p* < 0.10; ^**^p < 0.01; ^***^p < 0.001.

Coeff., coefficient; SE, standard error; VR, virtual reality.

**FIG. 3.**
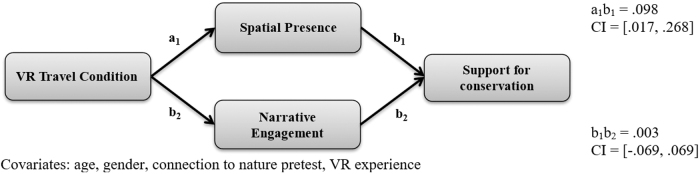
Indirect effect of VR travel condition on support for conservation.

Second, a parallel mediation analysis examined support for conservation (Table 1 and Fig. 3). The analysis found that VR travel condition positively predicted spatial presence (*b* = 0.80, *p* < 0.001) and narrative engagement (*b* = 0.50, *p* < 0.01). Spatial presence (*b* = 0.12, *p* < 0.05) then predicted students' support for conservation, while narrative engagement (*b* = 0.01, *p* = 0.87) did not. The indirect effect from VR travel condition to spatial presence and then to support for conservation was statistically significant (95% CI [0.017–0.268]). The indirect effect from VR travel condition to narrative engagement and then to support for conservation was not statistically significant (95% CI [−0.069 to 0.069]). Therefore, H3b was partially supported. The mediation model explained a statistically significant proportion of variance in support for conservation scores [*R*^2^ = 0.85, *F*(8, 57) = 41.00, *p* < 0.001].

**FIG. 4.**
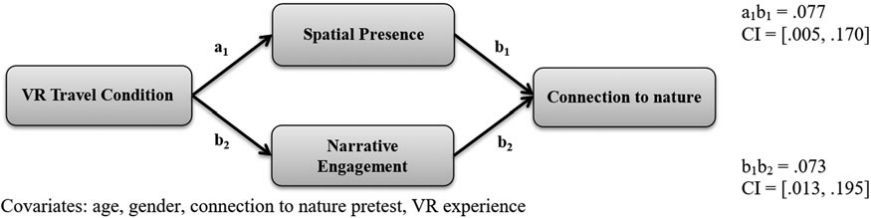
Indirect effect of VR travel condition on activism intentions.

Third, a parallel mediation analysis examined students' activism intentions (Table 2 and Fig. 4). VR travel condition positively predicted spatial presence (*b* = 0.86, *p* < 0.001) and narrative engagement (*b* = 0.54, *p* < 0.01). Spatial presence was a statistically significant predictor of activism intentions (*b* = 0.15, *p* < 0.05) while narrative engagement was approaching statistical significance (*b* = 0.13, *p* < 0.10). The indirect effect from VR travel condition to spatial presence and then to activism intentions was statistically significant (95% CI [0.005–0.310]). The indirect effect from VR travel condition to narrative engagement and then to activism intentions was also statistically significant (95% CI [0.003–0.194]). Therefore, H3c was supported. The mediation model explained a statistically significant proportion of variance in activism intention scores [*R*^2^ = 0.83, *F*(8, 57) = 33.77, *p* < 0.001].

**Table 2.** Mediation/Regression Results Showing Direct and Indirect Effects of Virtual Reality Travel Condition on Activism Intentions and Oceanic Attitudes

	Activism intentions			Oceanic attitudes		
	Coeff.		SE	Coeff.		SE
Direct effects						
Spatial presence	0.86	^***^	0.19	0.80	^***^	0.21
Narrative engagement	0.54	^**^	0.18	0.49	^**^	0.18
Indirect effects						
Spatial presence	0.15	^**^	0.07	0.23	^**^	0.09
Narrative engagement	0.13	^+^	0.07	−0.06		0.09
*F*	33.77	^***^		14.43	^***^	
*R* ^2^	0.83			0.67		

*Note:* age, gender, race, VR experience, and pretest results were controlled.

*N* = 66; ^+^*p* < 0.10; ^**^p < 0.01; ^***^p < 0.001.

**FIG. 5.**
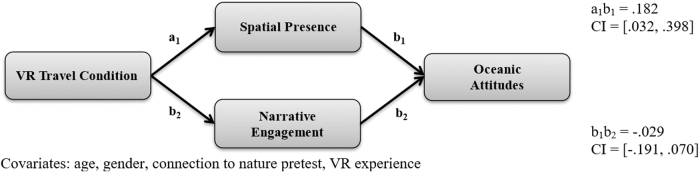
Indirect effect of VR travel condition on oceanic attitudes.

Finally, a parallel mediation analysis examined ocean protection attitudes (Table 2 and Fig. 5). The analysis found that VR travel condition positively predicted spatial presence (*b* = 0.80, *p* < 0.001) and narrative engagement (*b* = 0.49, *p* < 0.01). Spatial presence (*b* = 0.23, *p* < 0.01) then predicted students' ocean protection attitudes while narrative engagement (*b* = −0.06, *p* = 0.53) did not. The indirect effect from VR travel condition to spatial presence and then to ocean protection attitudes was statistically significant (95% CI [0.032–0.398]). The indirect effect from VR travel condition to narrative engagement and then to ocean protection attitudes was not statistically significant (95% CI [−0.191 to 0.070]). Therefore, H3d was partially supported. The mediation model explained a statistically significant proportion of variance in ocean protection attitude scores [*R*^2^ = 0.67, *F*(8, 57) = 14.43, *p* < 0.001].

## Discussion

The present study investigated the effects of nature-based VR travel and its potential to persuade people to be more environmentally connected and conscious. To do so, a theoretical framework was proposed that accounts for both the *context* and *content* of VR travel experiences. Specifically, the proposed theoretical framework hypothesized that the potential persuasive effects of nature-based VR travel would be mediated by spatial presence (i.e., context) and narrative engagement (i.e., content).

Results indicated no statistically significant difference between the VR travel condition and the TV control condition regarding participants' environmental attitudes and behavioral intentions. This finding is in line with previous research that found little difference between watching a nature-based 360° video in VR or on a tablet.^10^ However, while the nature-based VR travel experience did not appear to influence the environmental outcome variables directly, it did indirectly affect them through the mediating roles of spatial presence and narrative engagement. Alternatively, prior augmented reality research has found that there may be trade-offs between spatial presence and narrative.^28^ Therefore, the lack of a direct effect may be that spatial presence and narrative engagement canceled each other out. Future studies should include more elaborate study designs, such as 2 × 2 experimental designs, to explore further how these two concepts may interact during VR experiences.

Theoretically, nature-based VR travel experiences can positively impact crucial psychological mediator variables such as spatial presence and narrative engagement, which in turn affect various pro-environmental attitudes and behavioral intentions. Significantly, while many studies examine the importance of presence in VR, this study adds to our theoretical understanding of VR effects by including narrative engagement in the conceptual model. This study also demonstrates the need to explore first-person and participatory narratives when traveling in VR as persuasive mechanisms.

Future research should continue to explore the importance and impact of narrative engagement and spatial presence together when exploring VR effects. *For example*, scholars should examine if spatial presence and narrative engagement operate in a similar manner in other contexts, such as educational experiences. Likewise, VR scholars should examine the technical, psychological, and design factors that may foster spatial presence and narrative engagement. For instance, the ability to interact with virtual environments, or the story therein, may contribute to the development of spatial presence and narrative engagement.

Practically, this study is critical because we face increasing environmental disasters, which will necessitate changes in our travel habits. VR travel can reduce the negative environmental ramifications of travel by reducing peoples' carbon footprints while simultaneously improving their environmental attitudes and behavioral intentions. However, merely traveling in VR isn't sufficient to foster maximum positive impacts. Instead, VR experiences that promote feelings of being present in nature and engaged with a narrative appear to be best suited to promote positive change.

VR designers should consider potential factors that may improve users' sense of spatial presence and narrative engagement. *For example*, VR developers may wish to consider the technologically immersive factors such as intuitive controls. There is also a growing body of literature suggesting that VR can be harnessed for a myriad of prosocial functions.^8,9^ Results from this study indicate that nature-based virtual travel can positively influence pro-environmental attitudes. Therefore, organizations with an interest in pro-environmental messaging, such as conservation organizations, may wish to explore the possibilities of crafting “public service experiences” which leverage the affordances of VR for pro-environmental outreach.

### Limitations

Several limitations associated with the present study should be addressed in future research. First, the college student sample used in this study potentially limits the generalizability of the findings. Second, the length of the intervention was intentionally short with only a single exposure. Third, the relatively small sample size reduces the ability of this study to detect small effect sizes which may explain some of the nonsignificant findings of this study. Fourth, adjustments for multiple comparisons were not made during the data analysis. Finally, in this study both spatial presence and narrative engagement were analyzed as singular variables. Therefore, future studies should investigate nature-based virtual travel effects with a more diverse population, across multiple exposures, with larger sample sizes, adjustments for multiple comparisons, and should consider examining the subdimensions of spatial presence and narrative engagement.

## Conclusion

In sum, the current study found that nature-based VR travel had an indirect positive effect on many of the participant's environmental attitudes and behavioral intentions through the mediating roles of spatial presence and narrative engagement. In other words, feeling present in the virtual *context* and engaged with the virtual *content* resulted in the most significant positive impact. Therefore, nature-based VR travel may provide an avenue to make travel more sustainable and impactful.
